# Analysis of multiple single nucleotide polymorphisms closely positioned in the ovine *PRNP *gene using linear fluorescent probes and melting curve analysis

**DOI:** 10.1186/1471-2334-7-90

**Published:** 2007-08-03

**Authors:** David J French, Dominic Jones, David G McDowell, Jim A Thomson, Paul G Debenham

**Affiliations:** 1 LGC, Queens Road, Teddington, Middlesex, TW11 0LY, UK

## Abstract

**Background:**

Resistance and susceptibility to scrapie has been associated with single nucleotide polymorphisms located within codons 136, 154 and 171 of the ovine prion protein gene (*PRNP*). Dual-labelled HyBeacon probes were developed to analyse single and clustered polymorphisms within these and neighbouring codons.

**Methods:**

Extracted DNAs and unpurified blood samples were genotyped with respect to polymorphisms in *PRNP *codons 136, 141, 154 and 171. PCR amplicons were investigated using a LightTyper instrument, measuring the stability of probe/target hybridisation through peak melting temperatures and determining the sequence of nucleotides at polymorphic sites.

**Results:**

The performance of HyBeacon assays was evaluated in a validation study comparing genotypes with those obtained using a primer extension assay (Sequenom MassEXTEND) analysed on a MALDI-ToF mass spectrometer. Over 12,000 sheep samples were successfully genotyped, reliably detecting A^136^, V^136^, T^136^, T^137^, L^141^, F^141 ^R^154^, H^154^, L^168^, R^171^, Q^171^, H^171 ^and K^171 ^sequence variants using only 4 HyBeacon probes.

**Conclusion:**

HyBeacon assays provide an extremely robust and accurate method for the analysis of single and clustered *PRNP *polymorphisms in a high-throughput format. The flexibility of the diagnostic tests ensures that samples are correctly genotyped even in the presence of additional sequence variations that flank the polymorphisms of interest. Such sequence variations may also be neutralised using universal bases such as 5-nitroindole if required.

## Background

Scrapie, one of several transmissible spongiform encephalopathies (TSEs), is a neurodegenerative and fatal disease that affects sheep and goats. Scrapie is infectious and thought to be transmissible between animals via an isoform of the prion protein (PrP^sc^) [1]. The cellular prion protein (PrP^c^) is 256 amino acids in length and is encoded by the host prion protein gene (*PRNP*). Resistance and susceptibility to scrapie has been associated with single nucleotide polymorphisms (SNPs) affecting the amino acids specified by codons 136, 154 and 171 of the *PRNP *gene [2,3]. Five common alleles have been identified, commonly referred to by the single letter amino acid codes for these three codons (i.e. ARR, ARQ, VRQ, AHQ and ARH) [4]. TRQ and ARK alleles have also been described, arising from additional SNPs in codons 136 and 171 respectively [5,6]. Certain prion protein genotypes confer resistance to scrapie and selective breeding programmes have sought to increase the number of sheep that are genetically resistant and eventually eradicate the disease from sheep flocks [7] particularly via national screening programmes within Europe. In addition to classical scrapie, cases of atypical scrapie have recently been identified and linked to a polymorphism within codon 141 [8,9]. Genotypes such as ARR/ARR that are considered to be genetically resistant to classical scrapie appear to be susceptible to atypical scrapie infection [10-12].

Direct sequencing accurately genotypes samples with respect to SNPs in codons 136, 154 and 171 and will reliably identify any additional polymorphisms in neighbouring codons. However, genotyping technologies such as sequencing and restriction fragment length polymorphism (RFLP) analysis do not lend themselves to high-throughput analysis of large numbers of samples. High-throughput genotyping technologies benefit significantly from automated methods involving robotics and LIMS (laboratory information management systems) to track samples throughout the process. The analysis of large numbers of ovine samples has been performed using techniques such as fluorescent primer extension [13,14], ARMS (amplification refractory mutation system) [15] and MALDI-ToF (matrix assisted laser desorption ionisation time of flight) mass spectrometry [16]. Such technologies require multiple technical steps that are both time-consuming and costly. In contrast, homogeneous assays are performed in a single tube and typically reduce hands-on time and thereby the overall cost of the test. Homogeneous detection technologies frequently utilise the sequence specific hybridisation of fluorescent oligonucleotides and an associated property, such as the exonuclease dependent degradation of hydrolysis probes (also known as TaqMan), to monitor the real-time amplification of specific DNA sequences. Variations in probe hybridisation stability are exploited to differentiate closely related target sequences, such as *PRNP *polymorphisms [17].

Additional nucleotide polymorphisms have been identified in codons neighbouring 136, 154 and 171, with sheep possessing alleles such as AT^137^RQ, AR^143^RQ, AC^151^RQ, ARL^168^Q and ARQE^175 ^[18]. Whilst these haplotypes are relatively rare, the additional polymorphisms may compromise *PRNP *genotyping using primer extension and real-time PCR technologies since they will destabilise the hybridisation process and potentially cause miscalls.

We have developed homogeneous high-throughput diagnostic tests, employing the fluorescent HyBeacon^® ^probe technology [19,20], to reliably genotype sheep with respect to polymorphisms within codons 136, 154 and 171 polymorphisms in a background of neighbouring and potentially interfering SNPs and mutations. Analysis of the leucine/phenylalanine polymorphism within codon 141 [9] is also described.

Probes are included in PCR assays and emit greater amounts of fluorescence when hybridised to complementary target sequences than when single-stranded. Following amplification, the presence and identity of target sequences is determined by melting curve analysis. Dual-labelled probes, possessing two fluorescent dye labels attached to internal nucleotides, exhibit considerably larger signal-to-noise ratios and melting peaks compared with single-labelled probes of identical sequence [21]. The stability and melting temperature of hybridised probes depends on the degree of homology between HyBeacons and their target sequences. Increasing the reaction temperature above the melting temperature (Tm) of the HyBeacon causes probe/target duplexes to dissociate and the amount of fluorescence emission to decrease. Sequences differing by as little as a single nucleotide may be detected and differentiated on the basis of melting peak Tm. Furthermore, multiple polymorphisms may be analysed using a single probe, enabling the presence of additional polymorphisms flanking *PRNP *codons 136, 141, 154 and 171 to be detected through distinct shifts in probe Tm. The analysis of extracted DNA and unpurified sheep blood samples is described here.

## Methods

### HyBeacon probes

The probes employed for the analysis of *PRNP *polymorphisms were synthesised by ATDBio Ltd (Southampton, UK) and were each labelled using two fluorescein dT monomers (Glen Research, Virginia, USA). Polymorphisms within codons 136, 154, 171 and 141 were investigated using the dual-labelled HyBeacons detailed in table 1.

**Table 1:** Primer and probe sequences employed for *PRNP *analysis

**Primer/Probe**	**Sequence (5'-3')**
136F2 forward primer	GCAGCTGGAGCAGTGGTAGG
171R2 reverse primer	GATGTTGACACAGTCATGCAC
136A2 probe	GGGAAGFG**C**CAFGAGCAGGC3
154H2 probe	GACCGFTACTAFC**A**TGAAAACATG3
171R6 probe	CAGTGGAFC**GG**TAFAGTAAC5AGAA3
141C3 probe	GAGCAGGCCT**C**TFATAC5FTTTGG3

F and 3 represent fluorescein dT and 3' phosphate respectively. Common SNPs within codons 136, 141, 154 and 171 are underlined. 5 represents the universal base 5-nitroindol employed to neutralise polymorphisms within codons 143 and 175.

### DNA extraction from sheep blood samples

8918 purified DNA samples were analysed using the HyBeacon method, comparing 136, 154 and 171 codon data with homogeneous MassEXTEND™ (hME) and MALDI-ToF (Sequenom, Hamburg, Germany) results obtained from the same sample set. The 141C3 probe was evaluated using a further 400 DNA samples, again comparing genotype calls with MALDI-ToF data. EDTA-blood samples were aspirated from sealed vacutainers (BD, Oxford, UK), transferring 50μl to 96 square well blocks (QIAGEN, Crawley, UK) using a Tecan Genesis RSP 150 robot (Tecan, Berkshire, UK). Blood samples were transferred to 91 plate wells, leaving 5 wells available for positive and negative controls. DNA extraction was performed using a Chemagen Chemagic magnetic separation module I robot and Chemagic DNA Blood100 kits (Auto Q Biosciences, Berkshire, UK), eluting DNA into 250μl of tissue culture water (Sigma Aldrich, UK) in 96 low-well plates.

### Analysis of unpurified blood samples

3663 unpurified blood samples were analysed using the HyBeacon methodology, comparing genotype calls with hME and MALDI-ToF data obtained from extracted DNA samples. 9μl of each blood sample was diluted in 710μl of tissue culture water using 96 deep well plates and a Tecan Genesis RSP 150 robot. Blood samples were transferred to 91 plate wells, leaving 5 wells available for positive and negative controls. Samples were mixed for 10 minutes using a Grant-bio PMS-1000 microplate shaker (Fisher Scientific, Leicestershire, UK).

### Polymerase Chain Reaction

PCR reaction volumes were 5μl, containing 1μl of purified DNA or diluted blood sample, 1× QIAGEN PCR buffer, 0.05μM forward primer, 0.5μM reverse primer (table 1), 0.25 units HotStarTaq polymerase (QIAGEN, Crawley, UK), 3 mM total MgCl_2_, 1 mM dNTPs (GE Healthcare, Amersham, UK) and 100 nM of HyBeacon probe. Asymmetric PCR was employed to generate an excess of the target strand such that probe hybridisation was favoured over annealing of amplified sequences. Four 96 well plates of extracted DNA or diluted bloods were analysed simultaneously. 500× PCR mastermixes were prepared such that sufficient reagents were available for robotic preparation of 384 well PCR plates. A Hamilton LabSTAR robot (Hamilton, Birmingham, UK) was employed to add 10μl of Chill-Out liquid wax (Bio-Rad Laboratories Ltd, Hertfordshire, UK), 4μl of PCR mastermix and 1μl of sample to Microseal white 384 well PCR plates (Bio-Rad). Each 384 well PCR plate contained 4 negative controls and 16 extracted DNA controls of known genotype (ARH/ARH, ARQ/ARH, ARR/ARH, VRQ/ARR, AHQ/AHQ, VRQ/ARH, ARH/AHQ, VRQ/AHQ, ARR/AHQ, VRQ/ARQ, ARQ/AHQ, ARR/ARR, ARQ/ARQ, ARR/ARQ & 2× VRQ/VRQ). Each PCR mastermix contained a single HyBeacon probe, such that three 384 well plates were employed to analyse SNPs in codons 136, 154 and 171.

Amplification of target sequences was performed using a 384 well Tetrad thermal cycler (MJ Research). The 136F2 and 171R2 primers detailed in table 1 were employed to amplify a *PRNP *target sequence of 198 bp, comprising codons 119 through to 185. Following an initial denaturation to activate the hotstart enzyme (95°C 15 minutes), targets were amplified using 35 cycles (or 40 cycles for diluted blood samples) comprising denaturation (95°C 15 seconds), primer annealing (55°C 30 seconds) and extension of products (72°C 30 seconds). Following amplification, reactions were incubated at 72°C for 2 minutes prior to a denaturation (95°C 1 minute) and cool (20°C 10 minutes).

### Melting curve analysis

PCR plates were heated from 35°C to 75°C, at 0.1°C per second, using a LightTyper instrument (Roche Diagnostics, Lewes, UK). Fluorescence emission was monitored continuously to measure the stability of probe hybridisation and determine the identity of amplified target sequences. Melting peaks were constructed by plotting the negative derivative of fluorescence with respect to temperature (-d(Fluorescence)/dT) on the y-axis) against temperature (x-axis). Samples were genotyped with the LightTyper software version 1.5 using positive control samples to define peak standards. Peak Tm and area data was exported from LightTyper software version 1.1 to generate an independent genotype call.

### Determination of *PRNP *genotype using HyBeacon melting peaks

Samples were genotyped using a combination of LightTyper software calls and exported peak data. The LightTyper software typically assigned genotypes to more than 95% of samples based on a set of defined standards employing reference control samples. Less than 5% of samples, for which the genotype was obvious by eye, required manual analysis when the LightTyper software assigned weak positive or unknown calls due to minor variations of melt curves from defined standards. A second, independent genotype call was obtained by exporting peak data into a Microsoft Excel spreadsheet that employed a combination of peak Tms and areas to genotype samples. Peak area ratios were employed to identify heterozygous samples that potentially possessed more than two copies of the *PRNP *gene [22,23]. A sample genotype was only reported if LightTyper/manual calls were concordant with exported spreadsheet data. Genotypes and rare sequence variants are presented using the notation described by Goldmann *et al. *[18].

Genotypes generated in HyBeacon tests were compared with hME and MALDI-TOF analysis performed in parallel. The hME assay employs MassEXTEND primers that anneal to target sequences adjacent to the polymorphism of interest. DNA polymerase is employed with a mixture of nucleotides and terminators to extend from the primer and through the polymorphic site. The resultant mass of the primer extension product is determined by mass spectrometry and is used to determine the sequence of nucleotides at the polymorphic site [16].

### Melting curve analysis with complementary oligonucleotides

The affect of rare polymorphisms on probe Tm was predicted using complementary oligonucleotides. 100 nM of each probe was hybridised to 200 nM of oligonucleotide homologue in QIAGEN PCR buffer and a total of 3 mM MgCl_2_. Microseal white 384 well PCR plates (Bio-Rad) were heated to 95°C for 1 minute and cooled to 20°C for 1 minute, prior to melting curve analysis using a LightTyper instrument, heating samples from 35°C to 75°C, at 0.1°C per second. Peak Tm data was determined using LightTyper software version 1.1.

## Results

Amplified targets were reliably detected and identified using the Tms of HyBeacon melting peaks. Probes exhibited ΔTms greater than 5°C, enabling reliable detection and differentiation of A^136^, V^136^, L^141^, F^141 ^R^154^, H^154^, R^171^, Q^171 ^and H^171 ^melting peaks using the LightTyper software. Samples that were homozygous at a given codon yielded a single peak, of defined Tm, whereas heterozygous samples yielded two melting peaks with characteristic heights and areas. The mean Tms observed for each probe peak are detailed in table 2. The major *PRNP *alleles were typically identified if peak Tms deviated from expected values by less than ± 2°C. The acceptance criteria employed for the detection and identification of Q^171 ^melting peaks were narrower than the mean Tm ± 2°C due to the neighbouring L^168 ^polymorphism. In each case, the acceptance criteria for the major *PRNP *alleles exceeded the mean ± 3 standard deviations, ensuring robust detection of melting peaks. Sequencing was performed for samples that yielded unexpected peak Tms or discordant HyBeacon/MALDI genotype calls. The detection of additional polymorphisms (e.g. T^137 ^and L^168^) was achieved if observed peak Tms deviated from expected mean Tms (table 2) by at least 1.9°C.

**Table 2:** Probe Tms and acceptance criteria

**Probe**	**Melting peak**	**Mean Tm**	**SD**	**N**	**Tm Acceptance Criteria**
136A2	A^136^	62.0°C	0.49	2228	60.0°C – 64.0°C
136A2	V^136^	53.2°C	0.36	214	51.2°C – 55.2°C
*136A2*	*T*^136^	*56.9°C*	*0.15*	*8*	*55.2°C – 58.9°C*
*136A2*	*AT*^137^	*59.5°C*	*0.19*	*7*	*59.0°C – 60.0°C*
154H2	H^154^	56.6°C	0.51	300	54.6°C – 58.7°C
154H2	R^154^	49.9°C	0.29	2171	47.9°C – 51.9°C
171R6	R^171^	55.4°C	0.39	1524	53.4°C – 57.4°C
171R6	Q^171^	49.8°C	0.31	1392	48.4°C – 51.8°C
*171R6*	*QE*^175^	*49.7°C*	*0.21*	*12*	*48.4°C – 51.8°C*
*171R6*	*L*^168^*Q*	*47.9°C*	*0.19*	*5*	*46.9°C – 48.4°C*
171R6	H^171^	44.8°C	0.27	168	42.8°C – 46.8°C
*171R6*	*K*^171^	*41.0°C*	*0.15*	*8*	*39.0°C – 42.8°C*
141C3	F^141^	53.9°C	0.19	39	51.9°C – 55.9°C
141C3	L^141^	45.0°C	0.14	6	43.0°C – 47.0°C

The mean Tms for each probe melting peak are presented, where N is the number of peaks investigated and SD is the standard deviation. The peak Tms for the major *PRNP *alleles were obtained from six 384 well plates prepared on different working days, thereby accounting for sample-to-sample and run-to-run sources of variation. Variant alleles exhibiting uncommon genotypes or additional SNPs are *italicised*. Acceptance criteria for these rare polymorphisms were estimated from the few samples analysed, minimising the potential for miscalling melting peaks.

### Codon 136

**Figure 1:**
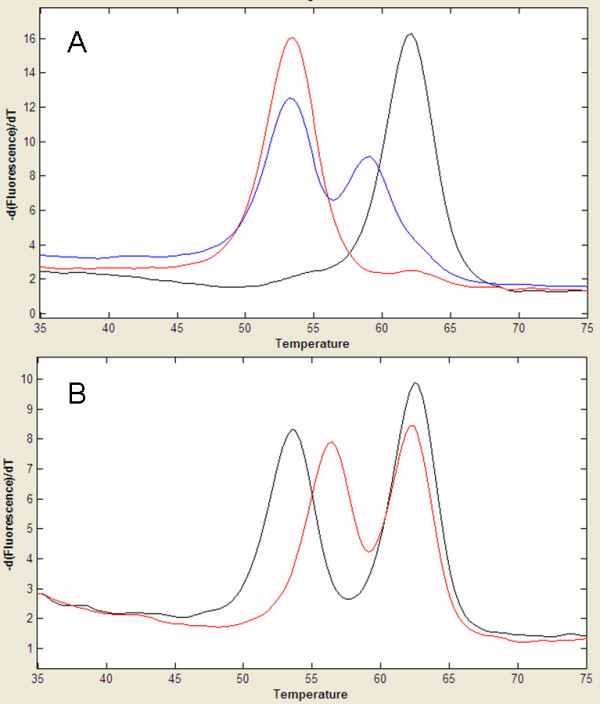
**Detection of additional polymorphisms with the 136 HyBeacon**. (A) The T^137 ^polymorphism is detected as a melting peak at approximately 59.5°C and is clearly distinct from the A^136 ^and V^136 ^peaks at 62.0°C and 53.2°C respectively. (B) T^136 ^is detected as a melting peak at approximately 56.9°C, enabling clear differentiation of genotypes such as ARR/VRQ and ARR/TRQ.

The C to T polymorphism in codon 136 (Ala [A]>Val [V]; GCC>GTC) was analysed using the HyBeacon 136A2, which possesses a C nucleotide at the polymorphic site. The probe generated a melting peak with a Tm of approximately 62.0°C when hybridised to the fully complementary alanine target sequence and exhibited a Tm of approximately 53.2°C when hybridised to the mismatched valine target sequence (Fig 1A). Samples that were heterozygous for A^136 ^and V^136 ^generated both 62.0°C and 53.2°C peaks (Fig. 1B).

A further T to C polymorphism within codon 137 (Met [M]>Thr [T]; ATG>ACG), with a frequency in the UK flock of approximately 0.012 [18], caused the 136A2 probe to generate a peak Tm of approximately 59.5°C (Fig. 1A). Samples possessing threonine at codon 137 were genotyped correctly and the polymorphism may either be included (e.g. AT^137^RQ/VRQ) or omitted (e.g. ARQ/VRQ) from reported genotypes. The presence of T^137 ^in 3 of the 8918 DNA samples was confirmed through sequencing.

An additional SNP within codon 136 is responsible for encoding threonine (Ala [A]>Thr [T]; GCC>ACC). The G to A polymorphism causes the 136A2 probe to exhibit a Tm of 56.9°C (Fig. 1B). This T allele has not been reported in studies of UK flocks [18], but has been detected in other countries [6]. Identification of the T^136 ^melting peak was demonstrated using Cypriot sheep samples of ARQ/TRQ and TRQ/TRQ genotype. Differentiation of V^136 ^and T^136 ^peaks was demonstrated using a mixture of VRQ/VRQ and TRQ/TRQ DNA samples. The 136A2 HyBeacon was demonstrated to be capable of simultaneously analysing at least three SNPs, generating four distinct melting peaks (table 2).

### Codon 154

The G to A polymorphism of codon 154 (Arg [R]>His [H]; CGT>CAT) was analysed using the HyBeacon 154H2, which possesses an A nucleotide at the polymorphic site. The probe generated melting peaks possessing an average Tm of 56.6°C when hybridised to the fully complementary histidine target sequence and exhibited a C/A mismatch and a Tm of approximately 49.9°C when hybridised to the arginine target sequence. Samples that were heterozygous for R^154 ^and H^154 ^generated both 56.6°C and 49.9°C peaks.

### Codon 171

Two adjacent SNPs present in codon 171 are responsible for encoding glutamine (CAG), arginine (CGG) and histidine (CAT). The two SNPs were simultaneously analysed using the HyBeacon 171R6, which yielded a peak Tm of approximately 55.4°C when hybridised to the fully complementary arginine codon. The probe exhibits single and double nucleotide mismatches when hybridised to glutamine and histidine sequence variants, reducing the Tm of hybridisation to approximately 49.8°C and 44.8°C respectively (table 2). Homozygous samples yielded single melting peaks and heterozygous samples generated two of the above peaks.

A rare SNP within codon 175 (Gln [Q]>Glu [E]; CAG>GAG) caused an earlier developmental version of the 171 HyBeacon to miscall ARR/ARQE^175 ^samples as ARR/ARH. This SNP has a reported frequency in the UK flock of 0.007 [18]. The E^175 ^allele reduced the Tm of the Q^171 ^peak such that it fell within the acceptance range of the H^171 ^peak. To prevent such miscalls during analysis, the polymorphism within codon 175 was neutralised using the universal base 5-nitroindole, which is a synthetic nucleotide analogue that hybridises equally with the four naturally occurring bases [24]. The 171R6 HyBeacon is unaffected by the E^175 ^SNP (table 2) and generates similar melting peaks with ARR/ARQ and ARR/ARQE^175 ^samples. An additional probe may be designed if analysis of E^175 ^is required.

A further SNP within codon 168 (Pro [P]>Leu [L]; CCA>CTA), with a reported frequency of 0.008 in the UK flock [18], was identified using the 171R6 HyBeacon. The C to T polymorphism within codon 168 was reliably identified through shifts in peak melting temperature, for example causing the Tm of the glutamine peak to be reduced to approximately 48°C. The L^168 ^polymorphism was detected in 17 purified DNA samples and 4 of the unpurified bloods. Samples possessing the L^168 ^SNP were genotyped correctly, confirmed by sequencing, and the polymorphism may either be included (e.g. ARR/ARL^168^Q) or omitted (e.g. ARR/ARQ) from reported genotypes.

An additional SNP within codon 171 is responsible for encoding lysine (Gln [Q]>Lys [K]; CAG>AAG). The 171R6 probe exhibits two nucleotide mismatches when hybridised to the lysine allele, generating a Tm of approximately 41°C. This K allele has not been reported in studies of UK flocks [18], but has been detected in other countries [5]. Identification of the K^171 ^melting peak was demonstrated using Cypriot sheep samples of ARR/ARK, ARQ/ARK and ARK/ARK genotype. Differentiation of H^171 ^and K^171 ^peaks was demonstrated using a mixture of ARH/ARH and ARK/ARK DNA samples.

The 171R6 HyBeacon is capable of simultaneously analysing four SNPs, generating five distinct peaks.

### Imbalanced melting peaks

Each parent sheep typically contributes a single *PRNP *allele to its progeny, such that maternally and paternally inherited alleles exist in a 1:1 ratio in 'normal' genotypes. However, a small subset of sheep have been found to exhibit abnormal (also known as complex) genotypes, where the two alleles of heterozygous samples appear to be present in unequal ratios [22,23]. Fluorescent primer extension, hME/MALDI-ToF and HyBeacon analyses have all yielded imbalanced peak data when analysing blood samples. The generation of imbalanced peak data may derive from a recognised genetic phenomenon known as chimerism or could be caused by the presence of additional polymorphisms in target sequences. Analyses employing alternate sample types, such as hair roots, ear tissue plugs and saliva, are expected to resolve a large proportion of the abnormal results obtained from blood.

Samples that yielded imbalanced melting peak data were called "Unknown" by the LightTyper software. Melting peaks were also determined to be imbalanced by measuring the ratio between mismatched and matched peak areas. Acceptance criteria for normal, balanced, heterozygotes were determined using the area ratios obtained from 4560 purified DNA samples (i.e. twelve 384 well plates, excluding negative controls). Acceptance criteria for peak area ratios (table 3) were determined using only A^136^/V^136^, R^154^/H^154^, R^171^/Q^171^, R^171^/H^171 ^and Q^171^/H^171 ^genotypes, excluding data generated in the presence of additional rare polymorphisms (e.g. R^171^/L^168^Q^171^). Samples that exhibit imbalanced melting peaks and area ratios that are outside of defined limits must be repeated for confirmation.

**Table 3:** Peak area ratios and imbalanced melting peaks

**Heterozygote**	**Mean area ratio**	**SD**	**N**	**Lower limit**	**Upper limit**
136 A/V	0.83	0.07	238	0.62	1.04
154 R/H	0.61	0.06	396	0.43	0.79
171 R/Q	0.97	0.08	1516	0.73	1.21
171 R/H	0.52	0.08	153	0.28	0.76
171 Q/H	0.75	0.06	46	0.57	0.93

4560 purified DNA samples were employed to determine the acceptance criteria for peak area ratios. The peak area ratios of heterozygous samples were calculated by dividing the area under the mismatched melting peak by the area under the matched melting peak. N is the number of heterozygous samples and SD is the standard deviation. Acceptance criteria for normal balanced heterozygotes were determined as the mean area ratio ± 3 standard deviations. Samples exhibiting imbalanced melting peaks generated area ratios outside of defined limits.

The estimated frequency of animals appearing to possess more than two copies of the *PRNP *gene is approximately 0.001 of the UK flock when analysing blood samples [22,23]. Our study of 8918 purified DNA samples was enriched for samples that had previously generated imbalanced peak data in MALDI-ToF analyses. Whilst 72 samples exhibited imbalanced MALDI peaks, only 43 heterozygous samples yielded imbalanced melting peaks.

### Assay performance

The performance of HyBeacon *PRNP *assays was assessed through determination of first time pass rates (FTPR) and concordance with parallel homogeneous MassEXTEND™ and MALDI-ToF analysis. Samples that contributed to the FTPR generated high quality peak data in 136, 154 and 171 assays. Samples were treated as "fails" if melting peaks were absent from any one of the tests or if software/analyst genotype calls were discordant with exported spreadsheet data. Samples were also excluded from FTPR if they exhibited imbalanced melting peaks. The average FTPR for 384 well plates was 97.8% (standard deviation = 1.23) with purified DNA samples.

HyBeacon and MALDI-ToF genotype calls were 99.8% concordant. The 17 DNA samples that yielded incongruent calls were sequenced and confirmed to possess leucine at codon 168, generating ARR/ARL^168^Q and ARL^168^Q/ARH genotypes in HyBeacon tests. These samples yielded imbalanced peaks in MALDI analyses and were reported as ARR/ARR/ARQ and ARQ/ARH/ARH respectively [22].

### Direct blood analysis

HyBeacon assays may also be performed directly using unpurified sheep blood samples. Blood samples only need to be diluted in water to reduce PCR inhibition and lighten sample colour enabling fluorescence detection. Five additional PCR cycles were required to analyse unpurified blood samples due to the low DNA concentrations once diluted. High quality melting peaks were obtained in 136, 154 and 171 assays, enabling accurate determination of sample genotypes. Analyses employing unpurified blood samples were extremely efficient yielding high quality melting peaks with the majority of samples, demonstrating first time pass rates between 85% and 95% (mean = 91.2%). Pass rates using unpurified blood were considerably more variable (standard deviation = 3.95) than PCR plates employing purified DNA samples. HyBeacon and MALDI-ToF data was 99.9% concordant, where the 4 DNA samples that yielded incongruent calls possessed leucine at codon 168. Blood samples yielded elevated numbers of weak positives with melting peaks of reduced height. Our study of 3663 unpurified blood samples was not enriched for samples that had previously generated imbalanced peak data in MALDI-ToF analyses. Whilst 7 samples yielded imbalanced MALDI peaks (with extracted DNA), 153 heterozygous samples exhibited imbalanced melting peaks in at least one assay.

### Predicting the affect of rare sequence variants

In addition to the T^136^, T^137^, L^168^, K^171 ^and E^175 ^polymorphisms described above, additional published polymorphisms in codons 138, 151, 154, 170 and 172 are covered by the diagnostic probes [14,18]. The affect of glycine at codon 170 (D170G; GAT>GGT) was investigated using two DNA samples previously genotyped as ARR/AHG^170^Q using a combination of hME, MALDI-ToF and sequencing analysis. The G^170 ^polymorphism reduced the Tm of the 171R6 melting peak to 40.0°C and is not expected to compromise the accuracy of *PRNP *genotypes. In the absence of suitable DNA samples, the affects of other rare polymorphisms were predicted by hybridisation of probes to complementary oligonucleotide sequences (table 4). Whilst melting peak Tms obtained with oligonucleotide and PCR targets are expected to vary (e.g. due to length and concentration differences), probe hybridisation to 200 nM of oligonucleotide homologue yielded melting temperatures that were comparable with PCR amplified target sequences.

**Table 4:** Predicted Tms of rare *PRNP *polymorphisms

**Probe**	**Polymorphisms**	**Oligonucleotide sequence (5'-3')**	**Mean Tm**	**SD**
136A2	A136	GCCTGCTCATG**G**CACTTCCC	61.6°C	0.13
136A2	V136	GCCTGCTCATG**A**CACTTCCC	53.3°C	0.13
136A2	A136 & S138R	GCCTGC**G**CATG**G**CACTTCCC	57.3°C	0
136A2	V136 & S138R	GCCTGC**G**CATG**A**CACTTCCC	47.3°C	0.13
136A2	A136 & S138N	GCCTG**T**TCATG**G**CACTTCCC	55.9°C	0.13
136A2	V136 & S138N	GCCTG**T**TCATG**A**CACTTCCC	44.9°C	0.18
154H2	H154	CATGTTTTCA**T**GATAGTAACGGTC	57.2°C	0.18
154H2	R154	CATGTTTTCA**C**GATAGTAACGGTC	50.2°C	0.18
154H2	H154 & R151C	CATGTTTTCA**T**GATAGTAAC**A**GTC	51.4°C	0
154H2	R154 & R151C	CATGTTTTCA**C**GATAGTAAC**A**GTC	43.1°C	0
154H2	H154 & R151H	CATGTTTTCA**T**GATAGTAA**T**GGTC	52.7°C	0.13
154H2	R154 & R151H	CATGTTTTCA**C**GATAGTAA**T**GGTC	45.2°C	0.13
154H2	H154 & R151G	CATGTTTTCA**T**GATAGTAAC**C**GTC	51.1°C	0
154H2	R154 & R151G	CATGTTTTCA**C**GATAGTAAC**C**GTC	42.8°C	0
154H2	R154L	CATGTTTTCA**A**GATAGTAACGGTC	52.2°C	0.18
171R6	R171	TTCTGGTTACTATA**CC**GATCCACTG	56.9°C	0
171R6	Q171	TTCTGGTTACTATA**CT**GATCCACTG	50.5°C	0.27
171R6	H171	TTCTGGTTACTATA**AT**GATCCACTG	45.2°C	0.18
171R6	R171 & Y172D	TTCTGGTTACTAT**CCC**GATCCACTG	50.5°C	0.28
171R6	Q171 & Y172D	TTCTGGTTACTAT**CCT**GATCCACTG	46.3°C	0.13
171R6	H171 & Y172D	TTCTGGTTACTAT**CAT**GATCCACTG	40.7°C	0.13
141C3	L141	CCAAAATGTATAA**G**AGGCCTGCTC	54.1°C	0.17
141C3	F141	CCAAAATGTATAA**A**AGGCCTGCTC	45.1°C	0.13
141C3	L141 & S138N	CCAAAATGTATAA**G**AGGCCTG**T**TC	49.4°C	0.13
141C3	F141 & S138N	CCAAAATGTATAA**A**AGGCCTG**T**TC	39.2°C	0.17
141C3	L141 & H143R	CCAAAA**C**GTATAA**G**AGGCCTGCTC	54.9°C	0.13
141C3	F141 & H143R	CCAAAA**C**GTATAA**A**AGGCCTGCTC	45.6°C	0.13

Complementary oligonucleotides were employed to predict the affect of rare polymorphisms on probe melting temperature. Six replicate analyses were performed for each target oligonucleotide. The mean Tm and standard deviation (SD) for melting peaks is presented. The nucleotides representing the polymorphisms in codons 136, 141, 154 and 171 are highlighted along with rare polymorphisms in neighbouring codons.

### Codon 141

A polymorphism within codon 141 has been linked to atypical scrapie [8,9,18]. Codon 141 falls outside the range of 136, 154 and 171 HyBeacons, so an additional probe was designed to analyse this common polymorphism. The C to T polymorphism of codon 141 (Leu [L]>Phe [F]; CTT>TTT) was analysed using the HyBeacon 141C3 (table 1), which possesses a C nucleotide at the polymorphic site. Melting curve studies with complementary oligonucleotides predicted that asparagine at codon 138 would be reliably detected along with L^141 ^and F^141 ^in all homozygous and heterozygous allele combinations (table 4). In contrast the R^143 ^polymorphism was predicted to cause L^141^R^143 ^alleles to be miscalled as F^141^. The R^143 ^polymorphism was therefore neutralised using the universal base 5-nitroindole as described above. Analysis of codon 141 was performed using the same primers employed for 136, 154 and 171 assays. The 141 HyBeacon generated a melting peak possessing a Tm of approximately 54°C when hybridised to the fully complementary leucine target sequence and exhibited a C/A mismatch and a Tm of approximately 44.5°C when hybridised to the phenylalanine target sequence. Samples that were heterozygous for L^141 ^and F^141 ^generated both 54°C and 44.5°C peaks. Sample genotypes were unaffected by the presence of arginine at codon 143, with HyBeacon and MALDI-ToF results in complete concordance.

## Discussion

The accuracy, reproducibility and robustness of HyBeacon assays was evaluated using in excess of 8900 purified DNA samples, comparing genotype calls with parallel homogeneous MassEXTEND™ and MALDI-ToF analysis. Samples were analysed in 384 well plates using a LightTyper instrument, achieving an average first time pass rate (FTPR) of 97.8%. HyBeacon and MALDI-ToF genotype calls were 99.8% concordant, where all discordant samples were sequenced and confirmed to possess leucine at codon 168. Fewer samples yielded imbalanced peak data in HyBeacon tests compared with hME and MALDI-ToF analysis of the same sample set, thereby reducing the number of samples requiring reanalysis.

Genotyping technologies that employ primer extension events to characterise downstream SNPs (e.g. SNaPshot, Applied Biosystems or hME, Sequenom) can be affected by the presence of additional polymorphisms within the primer sequences. Additional polymorphisms neighbouring the SNPs of interest can considerably reduce the stability of genotyping primers and affect the efficiency of extension, resulting in imbalanced peaks or allele drop-out and potentially incorrect calls. The presence of leucine at codon 168 reduces the efficiency of primer extension with L^168^Q alleles in Sequenom hME assays, creating MALDI peak imbalances in ARR/ARL^168^Q and ARL^168^Q/ARH heterozygotes. L^168 ^is detected using HyBeacons as a decrease in Tm, such that Q and L^168^Q targets may be reliably differentiated.

Alternate fluorescent probe technologies (such as hydrolysis probes), which rely on real-time fluorescence increases to detect and differentiate polymorphic sequences, will also be affected by the presence of additional SNPs in target sequences. Such real-time PCR technologies typically employ a separate probe for each sequence variant and would require seven probes just to analyse the common 136, 154 and 171 alleles [25]. The presence of additional SNPs within target sequences would decrease the stability of real-time probes, reducing or preventing the generation of fluorescent signal and potentially generating incorrect genotypes. The stability of HyBeacon hybridisation is also affected by the presence of rare polymorphisms. However, unlike the genotyping technologies described above, these additional SNPs are detected through decreases in peak Tm, generating novel melting peaks and correct calls. Post-amplification melting curve analysis of *PRNP *polymorphisms has also been presented for HybProbes that employ fluorescence resonance energy transfer (FRET) [26,27].

HyBeacon assays were able to detect the additional polymorphisms T^136^, T^137 ^L^168 ^and K^171 ^through distinct shifts in melting peak Tm. The rare polymorphisms R^138^, N^138^, C^151^, H^151^, G^151^, L^154 ^and D^172 ^[14,18], were not encountered during validation of the HyBeacon assays, but their effects were predicted using complementary oligonucleotide targets (table 4). R^138^, N^138^, H^151^, L^154 ^and G^170 ^are not expected to affect the accuracy of *PRNP *genotype calls since they result in melting peak Tms that are outside of the acceptance criteria established for A^136^, V^136^, R^154^, H^154^, R^171^, Q^171 ^and H^171 ^(table 2). C^151^, G^151 ^and D^172 ^polymorphisms are also expected to return the correct *PRNP *genotypes except when present in AHC^151^Q, AHG^151^Q, ARRD^172^, ARQD^172^, AHQD^172 ^and VRQD^172 ^alleles, of which only C^151 ^has been reported in the UK flock (at a frequency of 0.002) [18]. Further unpublished sequence polymorphisms could also give rise to incorrect genotype calls if they result in melting peaks that coincide with the expected Tms detailed in table 2. Several of the other reported polymorphisms, such as S^167 ^and K^176 ^fall outside the HyBeacon binding sites and have no effect on peak Tms.

All genotyping technologies have the potential to miscall *PRNP *genotypes when certain rare sequence variants are present in primer/probe sequences. These miscalls may remain undetected unless all samples are sequenced or genotypes compared with an independent genotyping method. Undesirable polymorphisms, which have the potential to generate incorrect calls, may be neutralised using universal nucleosides such as inosine, 3-nitropyrrole and 5-nitroindole [24], as described here with R^143 ^and E^175^. Alternatively, degenerative bases such as dP and dK phosphoramidites may be introduced into oligonucleotides to base pair with A and G or with C and T respectively (Glen Research, Virginia, USA). A more cost effective method to "mask" additional polymorphisms might be to design probe sequences such that they comprise a mismatched nucleotide at the polymorphic site when hybridised to all possible target alleles [28]. The Tm for each sequence variant should ideally differ by less than 1°C to ensure detection of further polymorphisms that may flank the masked position.

The 136, 141, 154 and 171 assays were performed in separate PCR plates, each containing a single HyBeacon probe. The HyBeacon assays were not combined into multiplexes to ensure that additional SNPs and imbalanced melting peaks were reliably detected. The bottle-neck for analysis within our laboratory was the number of PCR plates that could be prepared per day. However, with two Hamilton LabSTAR robots in operation up to 1440 samples could be analysed each day using 136, 154 and 171 assays. Furthermore, since each of the HyBeacon tests employs the same primer pair, a single PCR plate can be prepared for each batch of samples, without inclusion of probes or Chill-Out liquid wax. Daughter plates prepared robotically post-amplification will mix a portion of the target amplicon with a specific HyBeacon probe. This approach considerably increases the number of samples that may be analysed each day. Melting curve analysis using a LightCycler 480 instrument (Roche Diagnostics) or similar real time PCR system would negate the requirement for Chill-Out liquid wax. The LightCycler 480 instrument will also permit colour-multiplexing with 384 well plates, enabling multiple probes labelled with spectrally distinct dyes to be employed simultaneously in each well [19,27]. HyBeacon assays may be performed in both high-throughput and ultra-rapid formats, where assays performed in glass capillaries using a LightCycler instrument are completed within 30 minutes.

The accuracy, reproducibility and robustness of blood assays was evaluated using in excess of 3600 unpurified samples. Whilst the average pass rate was in excess of 90%, the increased analysis times and repeat rates required for direct blood analyses negate much of the cost savings achieved through omitting DNA extraction. Further development is required to enable unpurified blood samples to be analysed in a high-throughput scenario. However, "pen-side" tests could be performed using unpurified blood samples and a portable PCR instrument.

## Conclusion

The fluorescent probe assays described here provide an extremely accurate and robust method for the analysis of single and clustered *PRNP *gene polymorphisms. Target sequences were detected through the generation of melting peaks and differentiated on the basis of melting temperature. Genotyping assays were successfully validated through comparison with a MALDI-ToF method and DNA sequencing. The flexibility of the diagnostic tests enabled samples to be correctly genotyped in the presence of additional sequence variations flanking the polymorphisms of interest.

## Competing interests

The author(s) declare that they have no competing interests.

## Authors' contributions

DF designed the project and protocols involved, designed primers/probes, performed sample analysis and prepared this manuscript. DJ performed robotic preparation of PCR plates, assisted with sample analysis and compared genotypes with MALDI-ToF data. DM, JT and PD participated in the design of the project. JT contributed to preparation of the manuscript. All authors read and approved the final manuscript.

## Pre-publication history

The pre-publication history for this paper can be accessed here:


